# A terpenoid phytoalexin plays a role in basal defense of *Nicotiana benthamiana* against *Potato virus X*

**DOI:** 10.1038/srep09682

**Published:** 2015-05-20

**Authors:** Ran Li, Chuan-Sia Tee, Yu-Lin Jiang, Xi-Yuan Jiang, Prasanna Nori Venkatesh, Rajani Sarojam, Jian Ye

**Affiliations:** 1 Temasek Life Sciences Laboratory, National University of Singapore, Singapore 117604, Singapore; 2 State Key Laboratory of Plant Genomics, Institute of Microbiology, Chinese Academy of Sciences, Beijing 100101, China

## Abstract

Terpenoid phytoalexins function as defense compound against a broad spectrum of pathogens and pests in the plant kingdom. However, the role of phytoalexin in antiviral defense is still elusive. In this study, we identified the biosynthesis pathway of a sesquiterpenoid phytoalexin, capsidiol 3-acetate as an antiviral response against RNA virus *Potato Virus X* (PVX) in *Nicotiana benthamiana*. *NbTPS1* and *NbEAH* genes were found strongly induced by PVX-infection. Enzymatic activity and genetic evidence indicated that both genes were involved in the PVX-induced biosynthesis of capsidiol 3-acetate. *NbTPS1*- or *NbEAH*-silenced plant was more susceptible to PVX. The accumulation of capsidiol 3-acetate in PVX-infected plant was partially regulated by jasmonic acid signaling receptor COI1. These findings provide an insight into a novel mechanism of how plant uses the basal arsenal machinery to mount a fight against virus attack even in susceptible species.

Plants are faced with numerous biotic stresses throughout their lifespan. To overcome these challenges, plants have developed a series of efficient and versatile defense system such as system acquired resistance (SAR)^1^ and induced systemic resistance (ISR)^2^. Both systems have been well documented to recognize signals from pathogen or herbivore and activate various downstream signal transductions and ultimately lead to the biosynthesis of direct defensive proteins or compounds^1^,^2^. Although the signal perception and transduction during disease resistance signaling have been well-established, the mechanisms of how host-derived compounds kill or combat the pathogen especially at the beginning of the arm-race between host and pathogen are poorly understood. Among these host compounds, secondary metabolites such as terpenes and terpene-derived phytoalexin have been defined as a versatile defense arsenal of the plant against herbivores and microbes, although their mechanism of action is still unknown. The biosynthesis of terpenes takes place either in cytosol via mevalonic acid pathway or in plastid via methylerythritol phosphate pathway^3^. Terpenes, classified by the number of isoprene units (C_5_) in the molecule, are categorized into monoterpene (C_10_), sesquiterpene (C_15_), diterpene (C_20_), triterpene (C_30_) and so on. Due to the volatility of small monoterpenes and sesquiterpenes, they are well known to act as an aerial signal that repels herbivores or attracts nature enemy of herbivores^4^,^5^. The accumulation level of diterpenoid phytoalexin momilactone A in rice has high negative correlation with white-backed plant hopper (*Sogatella furcifera*) infestation, suggesting these phytoalexins are potential anti-herbivore compounds^6^. It has also been reported that (*E*)-β-caryophyllene directly inhibits the growth of bacteria *Pseudomonas syringae* pv. Tomato DC3000^7^. Additionally, capsidiol is the major phytoalexin produced in Solanaceae plants in response to fungus and bacterial infection. It is also involved in resistance to fungus *Botrytis cinerea* in *Nicotiana plumbaginifolia*^8^,^9^. Capsidiol is derived from farnesyl diphosphate by a two-step process catalyzed by 5-epi-aristolochene synthase (EAS)^10^ and 5-epi-aristolochene hydroxylase (EAH)^11^. Silencing of the homologous genes in *Nicotiana benthamiana* results in lower resistance to potato late blight oomycete^9^. In addition to fungi and bacteria, virus also poses serious threat to plants, causing major crop loss worldwide. However, to date only a few terpenoids have been characterized to participate in antiviral defense. A previous study reported that the diterpene WAF-1 acts as an endogenous signal that activates *tobacco mosaic virus* (TMV)-induced defense in *Nicotiana tabacum*^12^. When infected by TMV, capsidiol or capsidiol-3-acetate is produced in *N. tabacum* or *Nicotiana undulata* plants respectively, suggesting that these terpenoid phytoalexins may play a role in TMV resistance^13^,^14^.

In plants, two effective native antiviral pathways have been well identified, namely RNA silencing and plant innate immune response. RNA silencing pathway is conserved in higher plants and provide a basal but broad resistance to all viral pathogens^15^. Plant innate immunity was identified in specific host-virus pair(s) and confers extremely strong resistance to a specific kind of virus^16^. Some hormone pathways also play a role in basal defense against viruses. For example, exogenous application of jasmonic acid (JA) and then salicylate acid (SA) confers a broad spectrum of resistance to RNA viruses including TMV, *Cucumber mosaic virus* and *Turnip crinkle virus*^17^. JA signal pathway is activated upon binding of Ile-conjugated JA to its receptor COI1^18^, which has been proved to be involved in R gene-mediated antiviral defense^19^. However, the antiviral mechanism of JA signaling pathway remains elusive. The genes involved in basal defense against viruses that encode antiviral proteins or catalytic enzymes that synthesize secondary metabolites are largely unclear. Here, we identified a sesquiterpenoid phytoalexin capsidiol 3-acetate as a basal defense antiviral compound produced against RNA virus *Potato virus X* (PVX) in *N. benthamiana*. Its biosynthesis is catalyzed by NbTPS1 and NbEAH. Additionally the production of this phytoalexin is regulated by JA signal receptor COI1.

## Results

### *NbTPS1* and *NbEAH* are PVX-induced genes in *N. benthamiana*

**Figure 1:**
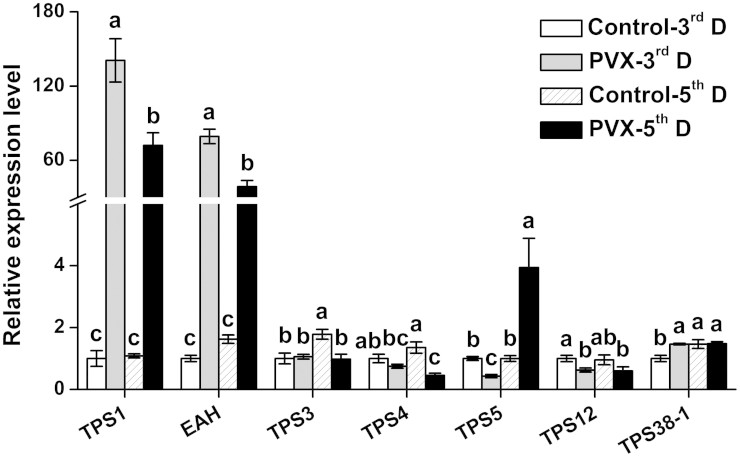
Expression of *terpenoid synthase* genes after PVX infection. Relative expression levels of different *terpenoid synthase* genes in third day/fifth day after treatment (3^rd^ D/5^th^ D) *N. benthamiana.* Plants were infiltrated with *Agrobacterium* carrying *Potato Virus X* (PVX) plasmid or pGreen empty vector alone (Control). Values are mean ± SE (n = 6). Letters indicate significant differences among different treatments (*P <* 0.05, Duncan's multiple-range test).

Recent studies of gene expression profiles in various pathosystems indicate that defense-related genes are expressed upon the infection of susceptible plants with several different viruses^20^,^21^, suggesting that susceptible plants recognize virus infection and do mount defense responses. As terpenoids like phytoalexins are transcriptionally regulated upon virus infection, we decided to check the expression levels of major terpene synthase genes in *N. benthamiana* after infection with positive-strand RNA virus PVX. Relative to mock infected leaves, the expression of monoterpene synthase, *NbTPS3* and *NbTPS4* were decreased after PVX-infection (Figure 1). Interestingly, the transcription levels of *NbTPS1* and *NbEAH* increased more than 50-times in PVX-infected leaves compared to uninfected leaves. In solanaceae plants, the *EAS* (homologs of *TPS1*) and *EAH* genes are associated with biosynthesis of terpenoid phytoalexin capsidiol or capsidiol 3-acetate, which are involved in pathogen-induced defense response^8^,^9^,^22^. Based on the reported sequences in *N. tabacum*, we cloned the full length mRNA of the two genes and named them as *NbTPS1* and *NbEAH* (NCBI ID number: KF990999 and KM410159). Few other *TPS*s were also mildly up-regulated or down-regulated by PVX infection. These results suggested that *NbTPS1* and *NbEAH* are the major terpene genes upregulated during PVX infection and might be involved in PVX-induced defense response.

### Silencing of *NbTPS1* and *NbEAH* attenuates plant resistance to PVX

**Figure 2:**
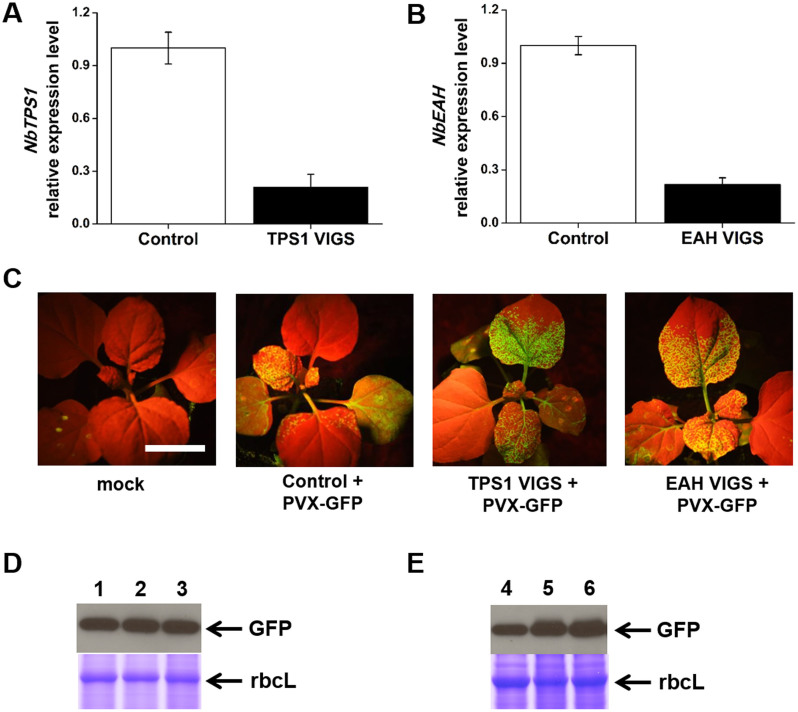
Silencing of *NbTPS1* and *NbEAH* decreases plant resistance against PVX. *sTRV1* and *sTRV2* vectors were used for *N. benthamiana* virus-induced gene silencing (VIGS). Ten days after inoculation, plants were further infiltrated with *Agrobacterium* containing PVX-GFP. (A) Relative expression level of *NbTPS1* gene in control and *NbTPS1-*silenced *N. benthamiana* plants. Values are mean ±SE (n = 6). (B) Relative expression level of *NbEAH* gene in control and *NbEAH-*silenced *N. benthamiana* plants. Values are mean ± SE (n = 6). (C) GFP imaging was performed under UV illumination 6 days after PVX-GFP infection. Mock, infiltrated with *Agrobacterium* only; Control, infiltrated with *Agrobacterium* containing *sTRV1* and empty *sTRV2* vector. Bar: 20 mm. (D) The amount of GFP in injected leaves. 1, Control plant; 2, TPS1 VIGS plant; 3, EAH VIGS plant. (E) The amount of GFP in systemic leaves. 4, Control plant; 5, TPS1 VIGS plant; 6, EAH VIGS plant. The large subunit of Rubisco (rbcL) is shown as a protein loading control. The experiment was repeated at least three times with similar results. Full-length blots/gels are presented in Figure S2.

To determine whether *NbTPS1* and *NbEAH* play roles in PVX resistance, we silenced them individually by Virus-Induced Gene Silencing (VIGS) followed by PVX infection (PVX-GFP, GFP overexpression viral vector). After VIGS, the expression levels of *NbTPS1* and *NbEAH* decreased by nearly 80% compared to control plants (Figures 2A and 2B). Silencing *NbEAH* did not affect the expression of two *NbEAH like* genes (*NbEAHL1* and *NbEAHL2*), which showed 66–84% nucleotide sequence similarity with *NbEAH* (Figure S1), indicating gene specific silencing in the *NbEAH* VIGS treated plants. The ability of plants to suppress PVX was measured by the fluorescence intensity or the amount of accumulated GFP in immunoblots detected by anti-GFP antibody. In comparison to control plants, *NbTPS1*- or *NbEAH*-silenced plants exhibited stronger GFP signal in systemic upper leaves (Figure 2C). Consistent results were also observed in immunoblot analysis, where the amount of GFP protein was higher in the systemic leaves of *NbTPS1*- and *NbEAH*-silenced plants than in that of control plants (Figure 2E). In contrast, the amount of GFP was similar between *NbTPS1*- or *NbEAH*-silenced plants and control plants in the local injected leaves (Figure 2D). These results indicated that *NbTPS1* and *NbEAH* genes were involved in *N. benthamiana* antiviral pathway.

### Epi-aristolochene and capsidiol 3-acetate are PVX-induced organic compounds in *N. benthamiana*

**Figure 3:**
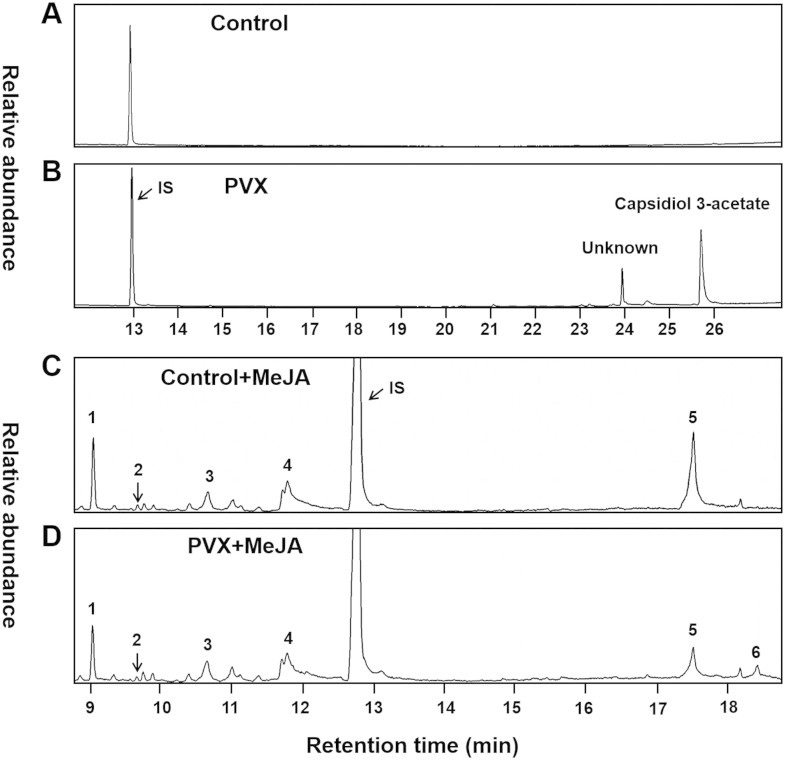
Altered volatile and non-volatile organic compounds by PVX infection. (A) Chromatogram of non-volatile compounds produced by *N. benthamiana* leaves that were infiltrated with *Agrobacterium* only*.* (B) Chromatogram of non-volatile compounds produced by *N. benthamiana* leaves that were infiltrated with *Agrobacterium* containing PVX*.* Chromatogram of volatile compounds emitted from *N. benthamiana* plants (C) and PVX-infected *N. benthamiana* plants (D) that were treated with methyl jasmonate (MeJA). IS, internal standard (camphor); 1, α-pinene; 2, β-pinene; 3, D-limonene; 4, linalool; 5, α-bergamotene; 6, epi-aristolochene.

To identify the compounds produced in response to PVX infection, we collected volatile and non-volatile organic compounds produced by *N. benthamiana*. However, no constitutive volatiles from headspace of *N. benthamiana* can be detected with our experimental equipment and conditions. This could be because of the minimal release of those compounds or because of its inducible characteristic. Therefore, we primed plants with methyl jasmonate (MeJA). And as a result, many terpenes could be detected after MeJA treatments (Figure 3C). We observed that PVX-infected-plants presented a different volatile profile compared to healthy plants (Figure 3D). The release of two monoterpenes α-pinene and linalool and a sesquiterpene α-bergamotene decreased in PVX-infected plants (Figure 3D; Figure S3). Strikingly, a novel sesquiterpene epi-aristolochene that was undetected in healthy plants was discovered in PVX-infected plant (Figure 3D, Figures S3 and S4). These results were consistent with the *TPS* gene expression profile after PVX-infection (Figure 1). It has been reported that the higher molecular weight terpenoid is produced by the epidermal cells of *N. benthamiana*^23^. Therefore, we hypothesized that the surface of *N. benthamiana* leaves may also produce some non-volatile terpenoid phytoalexins. Using hexane as a solvent for extraction, no compound could be detected from healthy leaves (Figure 3A), whereas two compounds were detected and identified in PVX-infected leaves. One of them was the sesquiterpenoid phytoalexin capsidiol 3-acetate (Figure 3B; Figure S5).

### Capsidiol 3-acetate is synthesized by NbTPS1 and NbEAH

**Figure 4:**
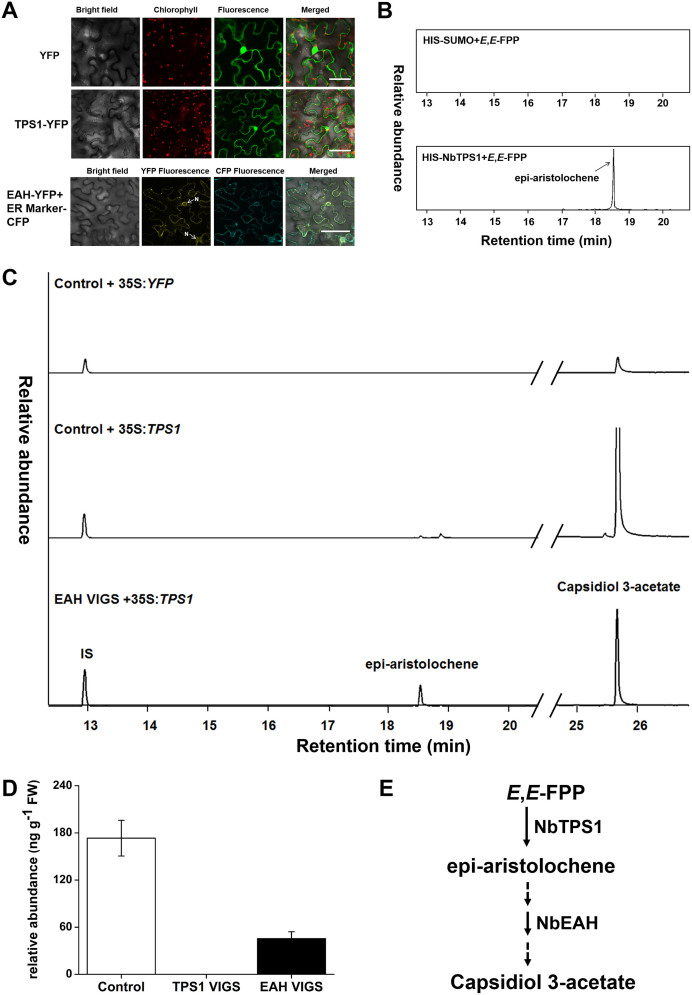
Capsidiol 3-acetate is synthesized by NbTPS1 and NbEAH. (A) Subcellular localization of NbTPS1 and NbEAH. *N. benthamiana* were transformed with *Agrobacterium* carrying either *YFP*, *NbTPS1*-*YFP* or *NbEAH*-*YFP* and *ER Marker*-*CFP*. After 48 h incubation, the transformed cells were observed under a confocal microscope. N, nucleus. Scale bar, 50 μm. (B) *In vitro* enzymatic assays of NbTPS1. Chromatogram of the products obtained by incubating (*E*,*E*)-FPP with recombinant proteins HIS-SUMO or HIS-NbTPS1. (C) *In vivo* enzymatic assays of NbTPS1. *N. benthamiana* leaves were co-infiltrated with *sTRV1* and *sTRV2*-*EAH* to obtain EAH VIGS plants, while plant co-infiltrated with *sTRV1* and *sTRV2* served as a control. Chromatogram of the products in control *N. benthamiana* leaf that were infiltrated with *Agrobacterium* containing *YFP* (Upper) or *TPS1-YFP* (Middle) or in *EAH*-silenced plant leaves that were infiltrated with *Agrobacterium* containing *TPS1*-*YFP* (Below). IS, internal standard (camphor). (D) Relative amount of capsidiol 3-acetate in *TPS1*-, *EAH*-silenced and vector control plants. (E) Models of capsidiol 3-acetate biosynthesis in *N. benthamiana*.

Enzymatic activity revealed that capsidiol is synthesized by EAS and EAH in *N. tabacum*^11^. We hypothesized that PVX-induced capsidiol 3-acetate is also correlated with high expression levels of *NbTPS1* and *NbEAH* in *N. benthamiana*. To clarify, we first determined the subcellular localization and function of NbTPS1. Subcellular localization assay showed that NbTPS1 was a cytosol protein (Figure 4A), suggesting that it might be a sesquiterpene synthase^3^. To determine the enzyme activity of this putative sesquiterpene synthase, we purified the recombinant protein His-NbTPS1 from *E. coli* and performed *in vitro* enzymatic assay with substrate (*E*,*E*)-FPP. Expectedly, a major peak was detected and identified as epi-aristolochene by GC-MS analysis (Figure S4). In contrast, no compound was detected when a yeast Small Ubiquitin-like Modifier (SUMO) protein (HIS-SUMO) was used in a similar enzyme activity assay (Figure 4B). Like the subcellular localization of other cytochrome P450s which are involved in the hydroxylation of terpene^24^, we found that NbEAH was also localized in endoplasmic reticulum (ER). The NbEAH: YFP co-localized with an ER marker: CFP in *N. benthamiana* leaf cells (Figure 4A). We further conducted an *in vivo* enzyme assay by transient expression of *NbTPS1-YFP* in *N. benthamiana* leaf cells by agro-infiltration. The *NbTPS1*-*YFP*-expressing leaves were found to produce large amount of capsidiol 3-acetate (Figure 4C middle), while *YFP*-expressing control leaves were found to produce only small amount of capsidiol 3-acetate (Figure 4C upper). This basal induction of capsidiol 3-acetate might be due to the *Agrobacterium* infiltration. We further did transient expression of *NbEAH* by *Agrobacterium* infiltration to investigate the function of this protein. The amount of capsidiol 3-acetate increased by 68% in *NbEAH*-*YFP*-expressing leaves as compared to only *YFP*-expressing leaves (Figure S6). Interestingly, we found that the amount of capsidiol 3-acetate produced by expressing *NbTPS1*-*YFP* was reduced in *NbEAH*-silenced plants. Instead accumulation of another compound epi-aristolochene was contrastingly high in these *NbEAH*-silenced plants (Figure 4C bottom). We further measured the native amount of capsidiol 3-acetate in *NbTPS1*- and *NbEAH*-silenced plant after infection with PVX. The production was reduced by nearly 75% in *NbEAH*-silenced plant when compared to control plant. Nearly no capsidiol 3-acetate was detected in *NbTPS1*-silenced plant (Figure 4D). These results indicated that both NbTPS1 and NbEAH were involved in the biosynthesis of capsidiol 3-acetate, in which NbTPS1 catalyzed the first step producing epi-aristolochene and subsequent hydroxylization by NbEAH. Other acyltransferase(s) might also participate in the subsequent downstream enzymatic process to produce the final product capsidiol 3-acetate (Figure 4E). The amount of capsidiol 3-acetate produced was highly dependent on the function of *NbTPS1* and *NbEAH* in PVX infection, prompting us to presume that might be an antiviral compound in *N. benthamiana*.

### Production of capsidiol 3-acetate is regulated by *COI1*

**Figure 5:**
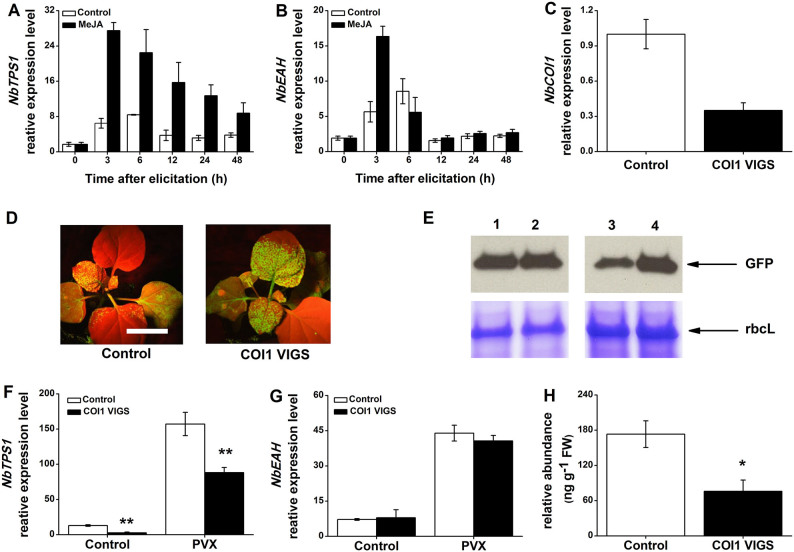
JA pathway is involved in the production of PVX-induced capsidiol 3-acetate. Relative *NbTPS1* (A) and *NbEAH* (B) expression levels (mean ± SE, n = 5) in *N. benthamiana* treated with MeJA or 0.01% Tween-20 (Control). (C) Relative expression level of *NbCOI1* gene in control and *NbCOI1*-silenced *N. benthamiana* plants. Values are mean ± SE (n = 6). (D) GFP imaging was performed under UV illumination 6 days after PVX-GFP infection. Mock, infiltrated with *Agrobacterium* only; Control, infiltrated with *Agrobacterium* containing *sTRV1* and empty *sTRV2* vector. Bar: 20 mm. (E) The amount of GFP in injected leaves (1, 2) and systemic leaves (3, 4) 1, Control plant; 2, COI1 VIGS plant; 3, Control plant. 4, COI1 VIGS plant. The large subunit of Rubisco (rbcL) is shown as a protein loading control. The experiment was repeated at least three times with similar results. Full-length blots/gels are presented in Figure S7. Relative *NbTPS1* (F) and *NbEAH* (G) expression levels (mean ± SE, n = 5) in COI1 VIGS and control *N. benthamiana* in sixth day after PVX infection. (H) Relative amount of capsidiol 3-acetate in COI1 VIGS and control plants. Values are mean ± SE (n = 6). Asterisks indicate significant differences between different treatments. (*, *P* < 0.05; **, *P* < 0.01; Student's *t*-test).

Jasmonic acid (JA) signal pathway plays a core role in regulation of terpene synthesis in plant^25^,^26^. We investigated if the synthesis of terpenoid phytoalexin, capsidiol 3-acetate, is also regulated by JA pathway. RT-qPCR analysis revealed that *NbTPS1* was significantly induced by MeJA treatment, whereas the transcription of *NbEAH* weakly increased after 3 h MeJA treatment (Figures 5A and 5B). To further confirm these genes were modulated by JA signaling pathway, we used VIGS to silence *NbCOI1*, a JA receptor, and tested the production of capsidiol 3-acetate and plant susceptibility to PVX. In the silenced plants, *NbCOI1* transcript levels were reduced by 65.0% (Figure 5C). NbCOI1 VIGS plant was more susceptible to PVX when compared to control plant (Figures 5D and 5E). *NbTPS1* expression was significantly repressed in *NbCOI*-silenced plant compared to control plant, but not *NbEAH* (Figures 5F and 5G). The reduced expression of *NbCOI1* resulted in diminished production of capsidiol 3-acetate (Figure 5H). Taken together, these results demonstrated that *NbCOI* mediated the production of capsidiol 3-acetate through regulating the transcription of *NbTPS1*.

## Discussion

Terpenes and terpenoids are natural products produced by a wide variety of plants. Since ancient times, mankind has used these compounds for healthcare. Terpene and its derivatives have broad medical application in human diseases, including antimicrobial, antifungal, antiparasitic and antiviral activity^27^. In plants, the antibacterial and antifungal activities of these compounds have also been characterized as well^7^,^8^,^28^,^29^. In this study, we genetically and biochemically identified that sesquiterpenoid phytoalexin, capsidiol 3-acetate, was involved in *N. benthamiana* defense against an RNA virus PVX. Capsidiol 3-acetate is synthesized by NbTPS1 and NbEAH. NbTPS1 catalyzes the main rate-limiting step, which is regulated by JA signal pathway. Our study provides the first genetic evidence indicating that sesquiterpenoid phytoalexin is regulated by JA and also involved in virus resistance. Unlike the effector induced immune resistance or RNA silencing, secondary metabolites terpene-based virus defense is milder but probably provide more broad-spectrum and persistent resistance to plants and most likely to animals as well. This type of basal defense is similar to plant pathogen-associated molecular patterns triggered immunity to recognize conserved patterns shared by several microbes, e.g. the bacterial flagellin^30^.

*NbTPS1* and *NbEAH* are significantly up-regulated by PVX infection (Figure 1), resulting in high levels of the sesquiterpenoid phytoalexin, capsidiol 3-acetate in PVX-infected leaves (Figure 3B). Results from our study support that capsidiol 3-acetate is synthesized by NbTPS1 and NbEAH. NbTPS1 converts (*E,E*)-FPP to epi-aristolochene (Figure 4B), which is the first step in capsidiol 3-acetate production. Transient expression of *NbTPS1* in *N. benthamiana* increased the amount of capsidiol 3-acetate (Figure 4C, middle). Contrastingly, no capsidiol 3-acetate could be detected in PVX-infected *NbTPS1*-silenced plants (Figure 4D). Compared to control plants, the production of capsidiol 3-acetate decreased significantly in *NbEAH*-silenced plants even with transient expression of *NbTPS1* (Figure 4C lower). Instead an accumulation of the intermediate product, epi-aristolochene was observed. Further transient expression of NbEAH alone was also sufficient to increase the production of capsidiol 3-acetate (Figure S6) and PVX-induced level of capsidiol 3-acetate was significantly reduced in *NbEAH*-silencing plants (Figure 4D). Additionally we found that *NbTPS1* transcription was regulated by NbCOI1-mediated JA signaling. Silencing of *NbCOI1* reduced the expression of PVX-induced *NbTPS1*, thereby decreasing the levels of PVX-induced capsidiol 3-acetate (Figures 5F and 5H). The capsidiol/capsidiol 3-acetate synthesis pathway is known to respond to various microbes including blight oomycete, fungal and virus^8^,^9^,^10^,^11^,^13^ (Figure 3B). Here, we also found that *Agrobacterium* injection can weakly induce the production of capsidiol 3-acetate (Figure 4C upper). Collectively, our study and previous research reveals that EAS- and EAH-mediated terpenoid phytolexin biosynthesis confers a broad resistance to microbial pathogens and viruses. In addition to NbTPS1 and NbEAH, other enzymes might also be involved in this proposed pathway. Using high throughput RNA sequencing of PVX-induced transcriptome, new genes in capsidiol 3-acetate biosynthesis pathway can be identified.

Many terpenes could be detected from headspace of MeJA-treated *N. benthamiana* (Figure 3C). However, epi-aristolochene could not be detected although *NbTPS1* is induced by MeJA treatment (Figure 5A). Interestingly, when (*Z*,*Z*)-FPP was used as the substrate in *in vitro* enzymatic assay, *NbTPS1* produced different sesquiterpenes, including α-bergamotene (Figure S8). It is possible that NbTPS1 might use different isoforms of FPP in response to different stresses, and the production of epi-aristolochene is in response to PVX or *Agrobacterium* infection (Figures 3D and 4C). The JA receptor COI1 is also involved in virus resistance (Figures 5D and 5E; Refs. 19, 31). Based on the regulation of PVX-induced NbTPS1 levels, we demonstrated that the terpenoid phytoalexin is one of the COI1-mediated defense responses (Figures 5F and 5H). But *NbEAH* was weakly induced by MeJA (Figure 5B) and was independent of COI1 (Figure 5G), suggesting that other signal pathways are also involved in the synthesis of terpenoid phytoalexin, e.g. ethylene or abscisic acid^8^,^9^.

Silencing of the biosynthesis of capsidiol 3-acetate pathway genes made plant more susceptible to PVX as indicated by increased accumulation of the GFP reporter protein. Based on our data, it can be postulated that the phytoalexin may function to inhibit plant virus systemic movement by affecting viral protein translation. We found no obvious changes in the PVX coat protein RNA levels in control and NbTPS1 silenced plants, indicating that capsidiol 3-acetate might function in regulating the virus post-transcriptionally (Figure S9). An antiviral compound *seco*-pregnane steroids from a well-known traditional Chinese medicine functions only in viral systemic movement but not in virus local infection^32^. The PVX systemic movement is regulated by various virus and host factors^33^. The capsidiol 3-acetate may have an affect on some specific step(s) of this virus-host interaction. For example, hydroxyl groups of capsidiol 3-acetate may interact with virus envelope lipids or inhibit viral attachment and cell penetration like other terpenes' function in animal cell^27^. Further experiments are needed to determine the exact antiviral mechanism of capsidiol 3-acetate.

In conclusion, we demonstrated that PVX-infection can activate the COI1 protein, which in turn increases the transcripts of *NbTPS1*. NbTPS1 convert (*E,E*)-FPP to epi-aristolochene, and which is then hydroxylated by NbEAH and probably catalyzed by other enzyme to produce the final product capsidiol 3-acetate. This terpenoid phytoalexin plays a role in PVX-related basal resistance. Disruption of its biosynthesis leads to higher susceptibility to PVX. Our finding provides a good example to illustrate basal defense against virus in susceptible plant species and enriches the plant antiviral theory.

## Methods

### Virus inoculation and GFP imaging

*N. benthamiana* plants with 4–6 true leaves were infiltrated with *Agrobacterium* carrying pGreen-PVX as described previously^34^. Infiltration with *Agrobacterium* carrying empty binary vector pGreen was used as controls. For virus-induced gene silencing (VIGS) plants, ten days after sTRV infiltration, the upper leaves were infiltrated with *Agrobacterium* carrying *PVX*-*GFP*. Six days after PVX injection, leaves were harvested for phytoalexin analysis (see below) or for GFP imaging as described^34^.

### Plant treatments

Four week-old *N. benthamiana* plants were sprayed with 100 μM methyl jasmonate (MeJA) (Sigma) containing 0.01% (v/v) Tween-20. After priming with MeJA treatment for 6 hours, plants were used for volatile analysis. Control plants were treated with 0.01% (v/v) Tween-20. Samples were collected at the indicated time points.

### Compound analysis

Collection, isolation and identification of volatiles from *N. benthamiana* plants were performed using the method as described previously^35^. Volatiles emitted from individual plant treated with MeJA were collected. The amount of compounds was expressed as percent of peak areas relative to the internal standard (camphor) per 18 h of trapping per group plants.

To isolate terpenoid phytoalexin produced in PVX-infected *N. benthamiana* leaves, two leaves (0.2–0.4 g) were dipped into 2 mL hexane (containing 2 mg internal standard camphor) in a 5 mL glass bottle and kept shaking for 5 min at room temperature. After centrifugation, the supernatant was transferred into a 2 mL GC vial and concentrated to 100 μL under a stream of nitrogen. The samples were then analyzed by using GC-MS.

### Constructs

Full-length open reading frames encoding *NbTPS1* or *NbEAH* without a stop codon were amplified by PCR using *Pfu* DNA polymerase (Thermo Scientific) with primers listed in Table S1. The DNA fragments were cloned either into pBA-YFP vector to generate GFP fused protein or pET28b (Novagene) to generate His-tag fused protein.

For VIGS experiments, partial sequences of *NbTPS1*, *NbEAH* and *NbCOI1* coding region were amplified using *Pfu* DNA polymerase (Thermo Scientific) with primers listed in Table S1. The DNA fragments were cloned into *psTRV2*^36^. Plasmids were introduced into *A. tumefaciens* AGL strain by electroporation.

### Quantitative RT-PCR

Total RNA was isolated using the RNeasy plant mini kit (Qiagen) and 800 ng of total RNA for each sample was reverse transcribed using the PrimeScript™ RT-PCR Kit (TaKaRa). Four to six independent biological samples were collected and analyzed. RT-qPCR was performed on an ABI 7900 HT fast real-time system (Life technologies) using SYBR Green Real-time PCR Master Mixes (Life technologies). The primers used for mRNA detection of target genes by RT-qPCR are listed in Table S1. The *N. benthamiana EF1α* mRNA was used as internal controls.

### Virus-induced gene silencing

Leaves of 3 week-old *N. benthamiana* plants were agroinfiltrated with *psTRV1* and *psTRV2*-*NbTPS1*, *psTRV2*-*NbEAH* or *psTRV2*-*NbCOI1* accordingly. Plants co-infiltrated with *psTRV1* and *psTRV2* were used as controls^36^.

### Subcellular localization

The vector containing *35S*: *NbTPS1*-*YFP* or *35S*: *NbEAH*-*YFP* was introduced into *A. tumefaciens* AGL1 strain by electroporation. *N. benthamiana* leaves were used to transiently express *NbTPS1*-*YFP*, *NbEAH*-*YFP* and *ER marker*-*CFP*^37^ by agroinfiltration. Two days after injection, YFP fluorescence was observed by using confocal microscope.

### *In vitro* and *in vivo* enzymatic assays of NbTPS1

The pET-28b vector containing full-length cDNA of *NbTPS1* was transformed into *Escherichia coli* BL21 (DE3). The expression was induced by adding 0.4 mM isopropyl-β-thiogalactopyranoside (IPTG) for 20 h at 20°C. Cells were collected and the recombinant protein was purified using His-Trap (GE healthcare) according to the manufacturer's instruction*. In vitro* enzymatic assays were performed in the following buffer conditions: 25 mM HEPES, 10% (w/v) glycerol, 5 mM DTT, 10 mM MgCl_2_, 100 μM (*E,E*)-FPP or (*Z*,*Z*)-FPP. 100 μg recombinant protein and incubated at 30°C for 2 h. The reaction was extracted with 500 μL of hexane and subjected to analysis by GC-MS. SUMO protein with His-tag was used as a control.

For *in vivo* enzymatic experiments, *N. benthamiana* transient expression system was used. The *Agrobacterium* containing *TPS1-YFP, EAH-YFP or YFP* alone and RNA silencing suppressor *Tomato bushy stunt virus* p19 were co-infiltrated into *N. benthamiana* leaves. Two days after injection, 0.4 g leaves were harvested and dipped in 2 mL hexane (containing 2 mg internal standard camphor) in a 5 mL glass bottle and kept shaking for 5 min at room temperature. After centrifugation, the supernatant was transferred into a 2 mL GC vial and concentrated to 100 μL under a stream of nitrogen. The samples were then analyzed by using GC-MS.

### Protein extraction and immunoblot

Six days after PVX-GFP infiltration, the injected leaves and system leaves were harvested. 0.15 g of each samples was extracted in 500 μL extraction buffer (50 mM Tris-HCl at pH 7.5, 150 mM NaCl, 2 mM MgCl_2_, 1 mM DTT, 20% glycerol) containing protease inhibitor cocktail (Roche). The cell debris was removed by centrifuging at 13000 rpm for 10 min. 2 μL of protein was separated by SDS-PAGE. After electrophoresis, the gels were stained with Coomassie Brilliant Blue or subjected to immunoblot analysis using anti-GFP antibody (Santa Cruz).

## Author Contributions

R.L. and J.Y. designed the experiments. R.L., C.S.T., Y.L.J., X.Y.J. and P.N.V. performed the experiments. R.L., R.S. and J.Y. analyzed data. R.L., R.S. and J.Y. wrote the article, which was reviewed and approved by all authors.

## Additional Information

**How to cite this article**:Li, R. *et al*. A terpenoid phytoalexin plays a role in basal defense of Nicotiana benthamiana against Potato virus X. *Sci. Rep.* **5**, 9682; doi: 10.1038/srep09682 (2015).

## Supplementary Material

Supplementary Information Supplementary Information
