# Supplementary material for: A terpenoid phytoalexin plays a role in basal defense of Nicotiana benthamiana against Potato virus X
Source: Sci Rep. 2015 May 20;5:9682. doi: 10.1038/srep09682 (PMC4438586; doi:10.1038/srep09682)
Supplement: Supplementary Information [file srep09682-s1.pdf]

Additional information

A terpenoid phytoalexin play roles in basal defense of *Nicotiana benthamiana* against *Potato virus X*

Ran Li[1*], Chuan-Sia Tee[1*], Yu-Lin Jiang[1], Xi-Yuan Jiang[1], Prasanna Nori Venkatesh[1], Rajani Sarojam[1], Jian Ye[1,2#]

[1] Temasek Life Sciences Laboratory, National University of Singapore, Singapore 117604, Singapore.

[2] State Key Laboratory of Plant Genomics, Institute of Microbiology, Chinese Academy of Sciences, Beijing 100101, China.

[*] Equal contribution

[#] Correspondence and requests for materials should be addressed to jianye@im.ac.cn

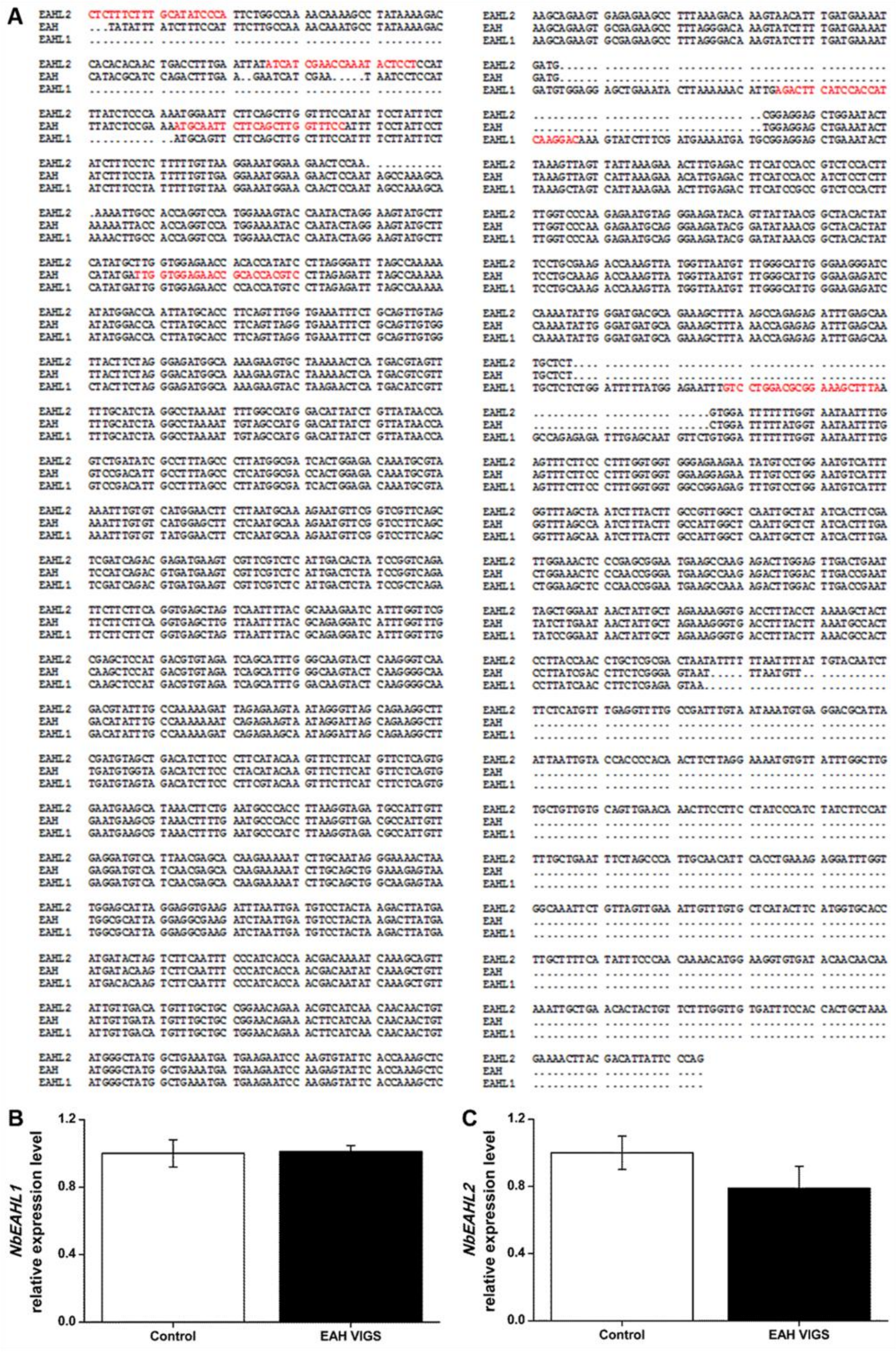

Figure S2. Silencing *NbEAH* does not affect the expression of two *NbEAH like* genes

(A) Alignment of *NbEAH* and *NbEAH like* sequences. Primers of each gene used for RT-qPCR analysis is labeled in red color. (B, C) Relative expression level of *NbEAHL1* (B) and *NbEAHL2* (C) genes in control and *NbEAH*-silenced *N. benthamiana* plants. Values are mean ±SE (n=7).

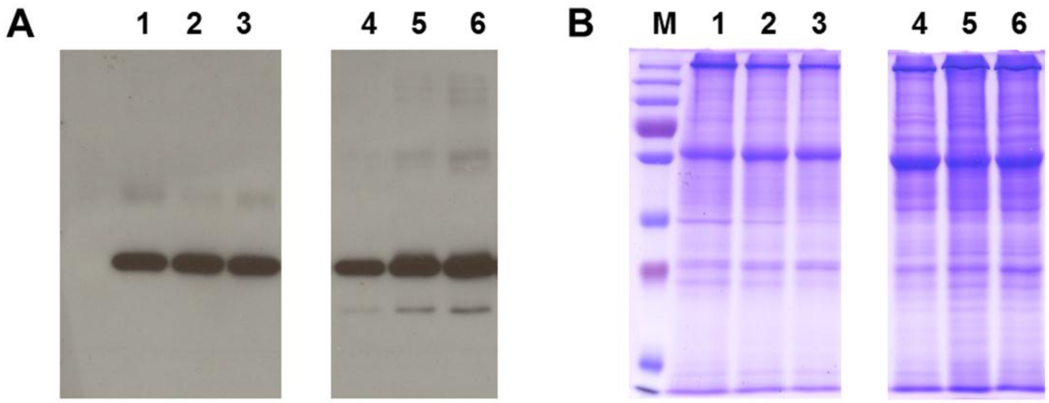

Figure S2. Full-length blots and gels of Figure 2 (D, E) in the main text.

(A) Immunoblot using anti-GFP antibody. (B) SDS-PAGE gel of protein loading. 1, Injected leaves of control plant; 2, Injected leaves of TPS1 VIGS plant; 3, Injected leaves of EAH VIGS plant; 4, Systemic leaves of control plant; 5, Systemic leaves of TPS1 VIGS plant; 6, Systemic leaves of EAH VIGS plant; Con, positive control; M, protein ladder.

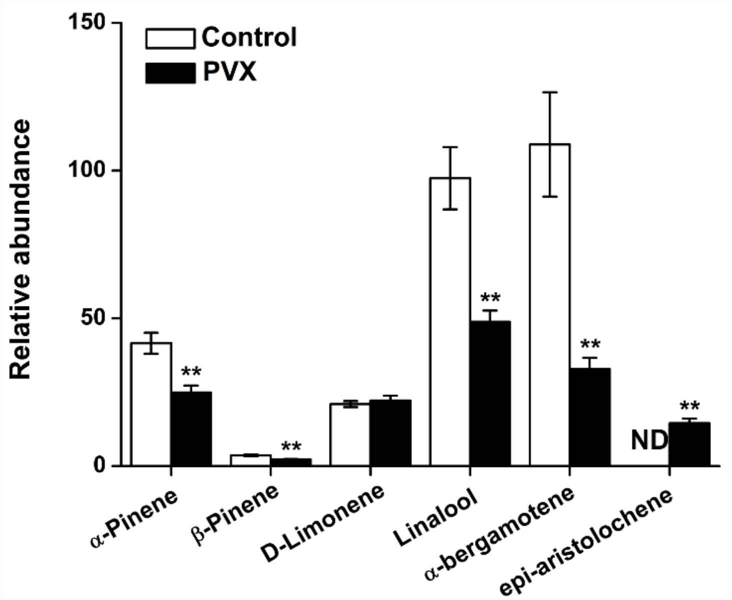

Figure S3.Terpenoids emitted by *N. benthamiana*after PVX infection.

Terpenes emitted by *N. benthamiana* after PVX infection. Values are mean relative amounts (% of internal standard peak area) ±SE (n=6). ND, not detected. Asterisks indicate significant differences between different PVX-infected plants and control plants. (**, P< 0.01; Student's *t*-test).

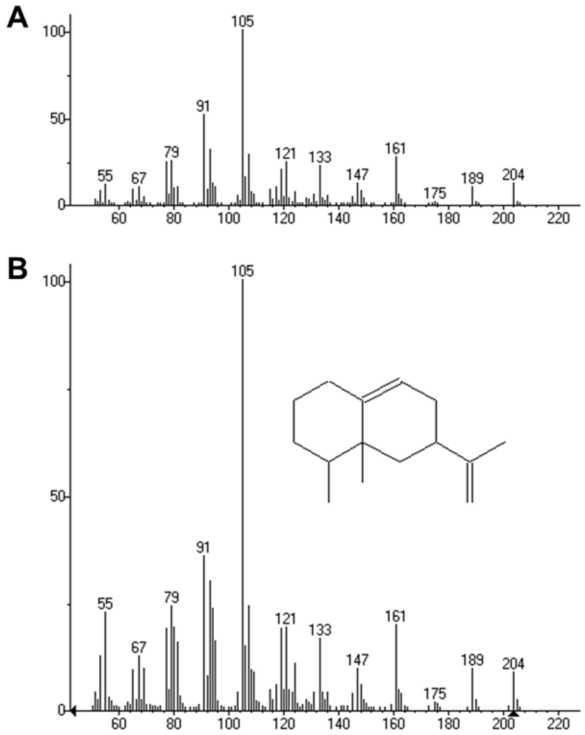

Figure S4. Mass spectra of the epi-aristolochene

(A). *In vitro* enzymatic reaction product of NbTPS1 by adding (*E*,*E*)-FPP as the substrate; (B). epi-aristolochene in NIST liberary.

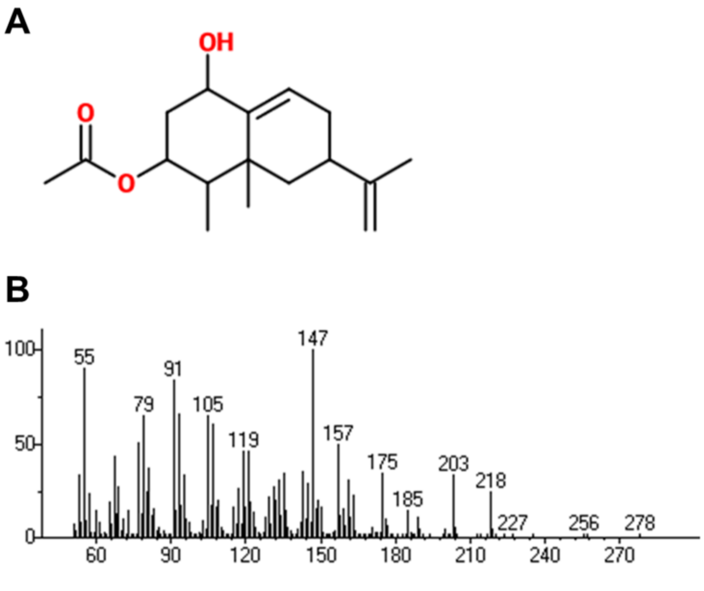

Figure S5. Chemical structure and mass spectra of the capsidiol 3-acetate

(A). Chemical structure of capsidiol 3-acetate; (B). capsidiol 3-acetate in NIST liberary.

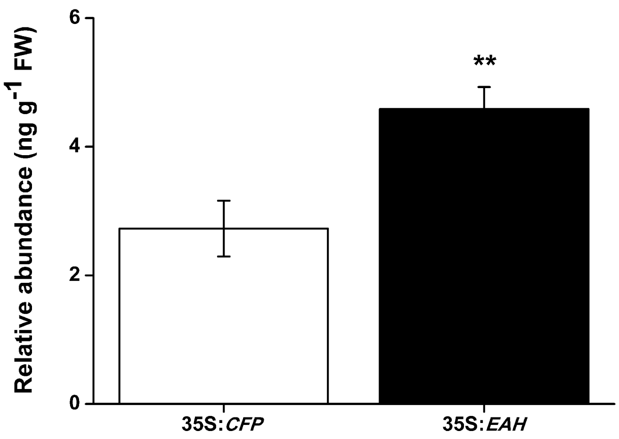

Figure S6. ***In vivo*** **enzymatic assays of NbEAH.**

N. benthamiana leaf that were infiltrated with *Agrobacterium* containing *YFP* or *EAH-YFP*. Relative amount of capsidiol 3-acetate in each plant was measured after two days of infiltration. Values are mean ±SE (n=6). Asterisks indicate significant differences between different plants. (**, P< 0.01; Student's *t*-test).

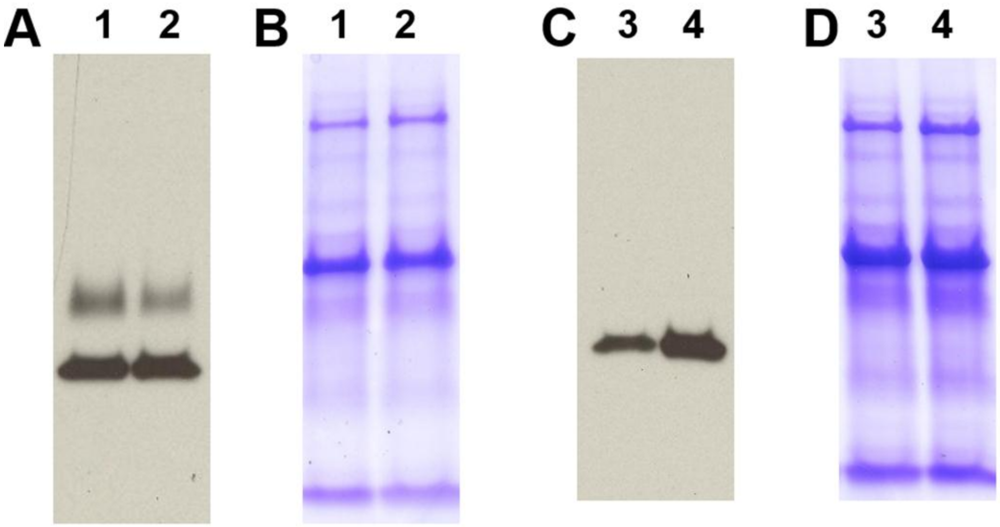

Figure S7. Full-length blots and gel of Figure 5 (E) in the main text.

(A, C) Immunoblot using anti-GFP antibody. (B, D) SDS-PAGE gel of protein loading. 1, Injected leaves of control plant; 2, Injected leaves of COI1 VIGS plant; 3, Systemic leaves of control plant; 4, Systemic leaves of COI1 VIGS plant.

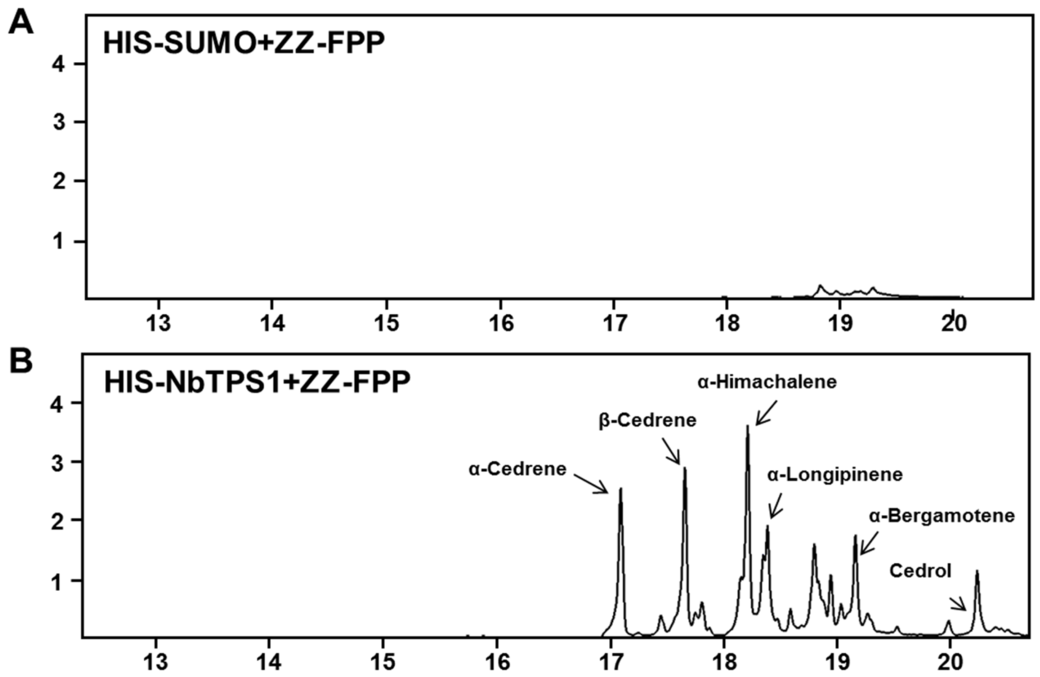

Figure S8. *In vitro* enzymatic assays of NbTPS1.

Chromatogram of the products obtained by incubating (*Z*,*Z*)-FPP with recombinant proteins HIS-SUMO or HIS-NbTPS1.

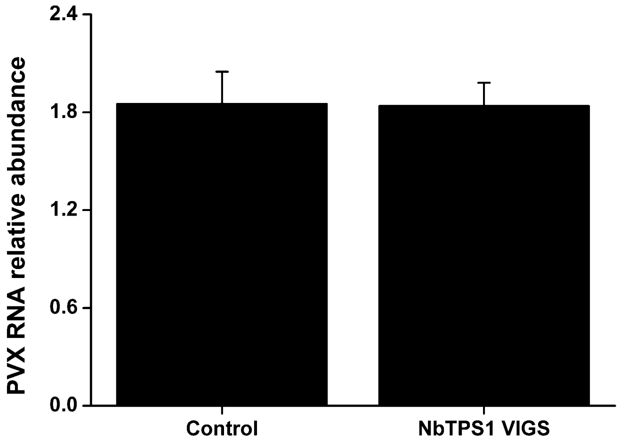

Figure S9. Relative expression level of PVX coat protein (CP) mRNA in different plants.

PVX RNA accumulation was measured by RT-qPCR and normalized to the level of *N. benthamiana EF1α* gene. Values are mean ±SE (n=8).

Table S1. **DNA primers used in this study.**

Gene	Sequence (5'-3')	Purpose
NbEAH-F	ATGCAATTCTTCAGCTTGGTTTCC	RT-qPCR
NbEAH-R	GACGTGGTGCGGTTCTCCACCAA	RT-qPCR
NbTPS1-F	TTAAAACGAACAAAAACAATACCCTCAT	RT-qPCR
NbTPS1-R	CTCTTGAGCATACATTTGTGCAACC	RT-qPCR
NbTPS5-F	ACATTGTTCAAGCAACACATCAAGAA	RT-qPCR
NbTPS5-R	CATCAAGAGTTGTAACAAGAGCATT	RT-qPCR
NbTPS3-F	TCACCAACCCAATTACGAAAGAGA	RT-qPCR
NbTPS3-R	CGTTTATCATTTTCCATGTCTCCT	RT-qPCR
NbTPS4-F	CGGATGAATTGAAGAGGGGTGATGTT	RT-qPCR
NbTPS4-R	ATGTGCTGTTCTTGCAATATTCTTT	RT-qPCR
NbTPS12-F	CGGCAGTGAACTTGATGAGA	RT-qPCR
NbTPS12-R	CAACTCCAACATGTGCTGCT	RT-qPCR
NbTPS38-1-F	ACGCAATAGAGCGACTACCTGACTA	RT-qPCR
NbTPS38-1-R	TGTTTGTTATCCATGCATTTCTCA	RT-qPCR
NbCOI1-F	GAGTTTGGTGGTGGCTCATT	RT-qPCR
NbCOI1-R	CCAAGTACGTCAAGCCCAAT	RT-qPCR
PVX-CP-F	GCACAAGGTTTCAAGCCTGA	RT-qPCR
PVX-CP-R	GCATCTAGGCTGGCAAAGTC	RT-qPCR
NbEAHL1-F	AGACTTCATCCACCATCAAGGAC	RT-qPCR
NbEAHL1-R	TAAAGCTTTCCGCGTCCAGGAC	RT-qPCR
NbEAHL2-F	CTCTTTCTTTGCATATCCCA	RT-qPCR
NbEAHL2-R	AGGAGTATTTGGTTCGATGAT	RT-qPCR
sTRV2-TPS1-F	ATAATTCTAGAGGCAACATGGTTTCAACATCTCTCCT	VIGS
sTRV2-TPS1-R	GTATGGATCCAAAATAAACTCCTAATGCCCAAAA	VIGS
sTRV2-NbEAH-F	AGGTAGGATCCGACATCTTCCCTACATACAA	VIGS
sTRV2-NbEAH-R	AGGTACTCGAGCATTTCAGCCATAGCCCATA	VIGS
sTRV2-COI1-F	TCCGCGAATTCCTTTACTAAGAGGTTGCTATAA	VIGS

sTRV2-COI1-R	TCAGTGGATCCAATGACTCAAGGACAAGTTA	VIGS
NbTPS1-YFP-F	AGTCTCCTAGGAATGGCCTCAGCAGCAGTAGC	localization
NbTPS1-YFP-R	AGTCGCCCGGGAAATTTGGATGGAGTCCACAA	localization
NbEAH-YFP-F	CGCCTCCTAGGAATGCAATTCTTCAGCTTGGT	localization
NbEAH-YFP-R	AGTAGCCCGGGA AGATAATTCGGTCAAGTC	localization
